# Predictive value and regulatory mechanism of serum miR-499a-5p on myocardial dysfunction in sepsis

**DOI:** 10.1186/s13019-021-01679-5

**Published:** 2021-10-15

**Authors:** Chuang Yang, Kun Wen

**Affiliations:** Department of Critical Care Medicine, The Second Hospital of Shandong University, No. 247 Beiyuan Dajie Street, Jinan City, 250012 Shandong Province China

**Keywords:** Sepsis, Myocardial dysfunction, miR-499a-5p, EIF4E, CK-MB, BNP

## Abstract

**Background:**

This study sought to investigate the predictive value and regulatory mechanism of serum miR-499a-5p in sepsis-induced myocardial dysfunction (SIMD).

**Methods:**

A total of 60 patients with sepsis and 60 healthy volunteers were enrolled in this study. The serum levels of miRNAs (miR-451, miR-378 and miR-499a-5p) were detected. Receiver operating characteristic curve and logistic regression analysis were used to evaluate the diagnostic and prognostic value of miR-499a-5p in SIMD patients. AC16 cells were used to establish SIMD model in vitro using lipopolysaccharide (LPS). An analysis was conducted for miR-499a-5p expression, cell viability, and the concentration of creatine kinase-MB isoform (CK-MB), brain natriuretic peptide (BNP), superoxide dismutase (SOD) and cytochrome C oxidase IV (COX IV). The downstream target of miR-499a-5p was verified.

**Results:**

Our results revealed a poor expression of miR-499a-5p in the serum of SIMD patients, while no significant difference was evident for miR-451 and miR-378. The level of miR-499a-5p in the survival group was higher than the non-survival group. miR-499a-5p elicited good diagnostic and prognostic value for SIMD. Our findings revealed that miR-499a-5p was decreased significantly in LPS-treated cardiomyocytes. After overexpression of miR-499a-5p, the cell viability increased, and the concentrations of CK-MB and BNP were decreased, while the concentrations of SOD and COX IV were increased. EIF4E was validated as the target of miR-499a-5p. After overexpression of EIF4E, the cell viability was decreased and the concentrations of CK-MB and BNP were increased while the concentrations of SOD and COX IV were decreased.

**Conclusion:**

The level of miR-499a-5p is weak in SIMD patients. miR-499a-5p has a good diagnostic and prognostic value for SIMD by inhibiting EIF4E transcription.

## Introduction

Sepsis is a systemic syndrome induced by an immediate response to infection or injury [1, 2]. Statically, sepsis is synonymous with high morbidity and mortality around the globe and poses an increasing burden on patients of all ages [3]. Fundamentally, sepsis is caused by pneumonia, abdominal, genitourinary and primary bacteremia, and infection on skin or soft tissues, and depending on the severity it could lead to cognitive and mental health impairment, intensive exacerbation of chronic medical conditions and other complications [4]. Currently, the most extensively adopted treatment for sepsis is restriction of infection via source control, antibiotics and organ function support [5]. An existing study determined that in most cases, sepsis would profoundly impact the function of lung, kidney and cardiovascular system [6]. Subsequent complicated intra-myocardial inflammation in sepsis could induce myocardial dysfunction [7]. Myocardial dysfunction is a prevalent complication of sepsis responsible for increased mortality in sepsis [8]. The current incidence of sepsis-induced cardiomyopathy is about 50% with 70% mortality [9]. However, currently no specific drug treatment could reverse the sepsis-induced myocardial dysfunction (SIMD).

microRNAs (miRNAs) are small RNA molecules with 19–25 nucleotides that function as regulators of certain target genes at the post-transcriptional level [10]. Existing research has identified the ability of miRNAs to participate in the regulation of sepsis by targeting the tumor necrosis factor, a primitive regulator of the pro-inflammatory process in sepsis, and the toll-like receptors which mediate systemic inflammation to pathogens in sepsis [11]. Several miRNAs can exercise vital therapeutic effects on sepsis. For instance, miR-15a could distinguish between sepsis and systemic inflammatory response syndromes [12]; miR-103a-3p alleviates sepsis-induced liver injury by targeting HMGB1 [13]; miR-21-3p modulates sepsis-induced cardiac dysfunction by regulating SORBS2 [14]; miR-499-5p overexpression mitigates cell apoptosis in lung tissues and represses inflammation in sepsis-induced lung injury [15]. Notably, miR-499a-5p can attenuate cardiomyocyte injury induced by hypoxia/reoxygenation (H/R) [16]. Therefore, we speculated the involvement of miR-499a-5p as a regulatory component in SIMD. An existing study highlighted the localization of eukaryotic translation initiation factor 4E (EIF4E) in the hearts of septic rats due to increased binding of 4E-BP1 and EIF4E [17]. However, limited literature has cited the functionality of miRNAs in cardiomyocytes in sepsis. The diagnostic or predictive value and molecular mechanisms of miR-499a-5p in myocardial dysfunction remain unidentified. The current study elucidated the diagnostic and prognostic value of serum miR-499a-5p in SIMD along with the regulatory mechanism.

## Materials and methods

### Ethics statement

The study protocol was conducted with approval of the ethics committee of The Second Hospital of Shandong University. The experiment procedures were compliant with the recommendations of the Declaration of Helsinki. All participants provided written informed consent prior to enrolment.

### Study subjects

A total of 60 patients with SIMD in the ICU of The Second Hospital of Shandong University from a period spanning over August 2016 to December 2017 were chosen for the current study. All patients were conclusively diagnosed in strict accordance with the diagnostic criteria of the International Sepsis Definition Conference [18]. The inclusion criteria were as follows: patients aged between 20 and 100 years with a diagnosis of sepsis or septic shock in the intensive care unit through the emergency department. In the case of no history of heart disease, the left ventricular ejection fraction (LVEF) of patients with sepsis less than 50% was regarded as the diagnostic criteria of myocardial dysfunction in sepsis [19]. The exclusion criteria were as follows: patients with immunosuppression; pregnant; human immune deficiency virus was positive; receiving immunosuppression, steroids or radiotherapy. Another group of 60 healthy people (healthy volunteers with similar age and gender distribution, no systemic inflammation, no history of tumor and no cardiac and renal dysfunction) were recruited as the control group with routine physical examination in the same hospital. Blood samples were withdrawn from patients with sepsis and myocardial dysfunction within 24 h of admission to ICU. Several clinical characteristics including age, gender, body mass index (BMI), and the levels of C-reactive protein (CRP), procalcitonin (PCT) and brain natriuretic peptide (BNP) were all documented.

### Sample preparation and total RNA isolation

The collected peripheral blood (about 5 mL) was transferred in a tube containing gel and clot activator for centrifugation for 15min at room temperature at 670 g. The supernatant was transferred to a new tube for repeated centrifugation at 3354 g for 30 min to remove cell debris. The supernatant was preserved at minus 80 °C with extraction of the RNA content.

The total serum RNA content was purified with the mirVana PARIS kit (Ambion, Grand Island, NY, USA) in strict accordance with the provided liquid sample protocol. All serum total RNA content was pretreated with DNase I (Promega, Madison, WI, USA) for 30 min to eliminate potential DNA contamination.

### Cell culture

Human cardiomyocyte AC16 cells provided by the cell bank of Chinese Academy of Sciences were cultured in DMEM (Invitrogen, Carlsbad, CA, USA) containing a combination of 100 U/mL penicillin, 100 mg/mL streptomycin (Invitrogen) and 10% fetal bovine serum (FBS) (GIBCO, NY, CA, USA) in a humidified incubator at 37 °C with 5% CO_2_. After transfection, the AC16 cells were treated with 10 μg/mL lipopolysaccharide (LPS) (Sigma, St. Louis, MO, USA) for 24 h for establishment of the sepsis-induced myocardial injury model in vitro.

### Cell grouping and transfection

The cells were divided into the control group, the LPS group, the LPS + mimic-NC group, the LPS + miR-499a-5p group, the LPS + miR-499a-5p + oe-NC group, and the LPS + miR-499a-5p + oe-EIF4E group. The plasmids of mimic NC, miR-499a-5p-mimic, pcDNA3.1-NC, pcDNA3.1-EIF4E were provided by Ribobio Co, Ltd. (Guangdong, China). The AC16 cells were seeded in 6-well plates at a concentration of 5 × 10^5^/cm^2^. After 24 h of culture, the AC16 cells were transfected with the aforementioned plasmids for 24 h using Lipofectamine 2000 (Invitrogen). The total RNA content was extracted using the RNA separation kit (Takara, Dalian, China). The transfection efficiency was estimated by means of reverse transcription quantitative polymerase chain reaction (RT-qPCR).

### RT-qPCR

After extraction of total RNA content, it was reverse transcribed into cDNA using an iScript^TM^ cDNA kit (Bio-Rad, Hercules, CA, USA). The RT-qPCR was conducted on ABI 7900HT real-time PCR system (ABI, Foster City, CA, USA) using the Takara SYBR supermix kit (Bio-Rad). The miRNA expression level was normalized in reference to U6. The gene expression was detected using the ABI 7900HT rapid real-time PCR system (ABI) and the SYBR-Green super mix kit (bio RAD). The primer sequences are shown in Table 1. GAPDH was regarded as loading control for the aforementioned experiment. The relative expression of genes was calculated based on the 2^-△△Ct^ method.

**Table 1:** Primer sequences of RT-qPCR

	Forward primer (5′–3′)	Reverse primer (5′–3′)
*miR-451*	GCCGAGCTTGGGAATGGC	CTCAACTGGTGTCGTGGA
*miR-378*	GCCGAGAGGGCTCCTGAC	CTCAACTGGTGTCGTGGA
*miR-499a-5p*	GCCGAGGCCCTGTCCCCT	CTCAACTGGTGTCGTGGA
*EIF4E*	GACTGTCGAACCGGAAACCA	AAACTTGGAGATCAGCCGCA
U6	CTCGCTTCGGCAGCACA	AACGCTTCACGAATTTGCGT
GAPDH	CAGTCACTACTCAGCTGCCA	GAGGGTGCTCC GGTAG

EIF4E, Eukaryotic initiation factor 4E; GAPDH, Glyceraldehyde-3-Phosphate Dehydrogenase

### Cell counting kit-8 (CCK-8) assay

After LPS treatment or transfection, the AC16 cells were incubated with the CCK-8 solution (Amyjet, Wuhan, China) at 37 °C and 5% CO_2_ for 4 h. After 0 h, 24 h, 48 h and 72 h, the absorbance value was measured at the excitation wavelength of 450 nm using a microplate reader (Molecular Devices, Shanghai, China).

### Enzyme-linked immunosorbent assay (ELISA)

According to the provided instructions, the CK-MB (creatine kinase MB, No. a12067), BNP (No. B20278), superoxide dismutase (SOD, No. J20542) and cytochrome C oxidase IV (COX IV, No. 0-023081) concentrations in AC16 cells were detected by means of ELISA kits (Shanghai Jianglai Biological Technology Co., Ltd, Shanghai, China).

### Bioinformatics

A combination of RNA22 [20], Starbase (http://starbase.sysu.edu.cn/) [21] and TargetScan (http://www.targetscan.org/vert_72/) [22] online websites were used to predict and screen the downstream target genes of miR-499a-5p and identify the corresponding binding sites.

### Dual-luciferase reporter gene assay

The EIF4E fragment containing miR-499a-5p binding site (WT) and mutant site (MUT) were constructed and subsequently planted into the pGL3 promoter plasmid (Promega). The constructed luciferase reporter plasmids were co-transfected with mimic-NC or miR-499a-5p-mimic into the AC16 cells respectively. After 48 h, the luciferase activity was detected using a luciferase assay kit (Beyotime, Shanghai, China).

### Statistical analysis

All statistical data analyses were processed using the SPSS 18.0 software (IBM Corp., Armonk, NY, USA) and GraphPad Prism 8.0 (GraphPad Software Inc., San Diego, CA, USA). The measurement data were presented as mean ± standard deviation. The T-test was adopted for comparison between two groups. One-way or two-way analysis of variance (ANOVA) was adopted for comparison among multiple groups, following Tukey’s post-hoc test. Receiver operating characteristic (ROC) and logistic regression analysis were used to evaluate the diagnostic and prognostic value of miR-499a-5p. In all statistical references, a value of *P* < 0.05 was considered to be statistically significant.

## Results

### Expression of miRNA in serum of SIMD patients

**Fig. 1:**
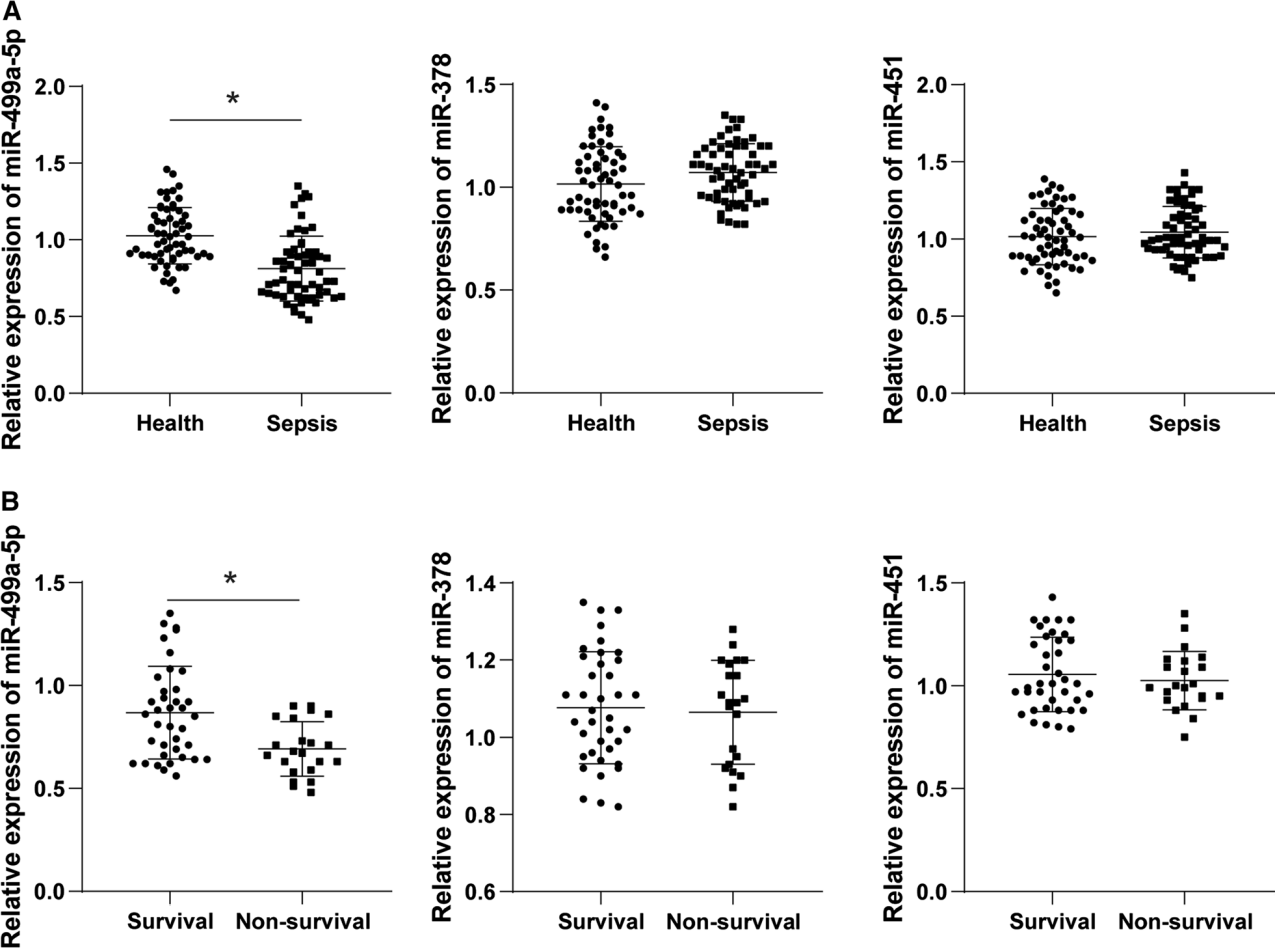
Expression of miRNAs in serum of SIMD patients. **a** The expression of miR-499a-5p, miR-378 and miR-451 was detected by RT-qPCR (N = 60); **b** The expressions of miR-499a-5p, miR-378 and miR-451 in survival group (N = 38) and non-survival group (N = 22) were compared; the *t* test was used in figure **a** and **b**, *P < 0.05

Table 2 summarizes the clinical characteristics of enrolled subjects. No significant difference was evident in parameters such as age, gender and BMI between healthy subjects and sepsis patients (*P* > 0.05), however the serum PCT, CRP and BNP levels in SIMD patients were higher than those in healthy participants (*P* < 0.05). To explore the predictive value of serum miRNAs on SIMD, we screened three miRNAs relevant with myocardial diseases according to the miRNAs predicted in a previous study [23]. Numerous studies have identified an association between miR-451, miR-378 and miR-499a-5p and myocardial diseases [16, 24–26]. Initially, a comprehensive analysis was conducted for the levels of serum miRNAs (miR-451, miR-378, miR-499a-5p) between the healthy group and sepsis group. According to RT-qPCR (Fig. 1a), compared with healthy controls, no significant difference was apparent in the serum levels of miR-451 and miR-378 in SIMD patients, while miR-499a-5p was significantly decreased (*P* < 0.05). Next, the SIMD patients were classified as the survival group and non-survival group. From a total of 60 patients with sepsis, 38 survived. Next, a comparison was conducted for the levels of miR-451, miR-378 and miR-499a-5p between the survival group and the non-survival group. Our results demonstrated an increased miR-499a-5p level in the survival group relative to the non-survival group (*P* < 0.05). Overall, our findings elicited a potential relationship between miR-499a-5p and the occurrence and prognosis of SIMD patients.

**Table 2:** Characteristics of clinical cases

Parameters	Healthy (N = 60)	Sepsis (N = 60)	*P* value
Age (years)	53.1 ± 10.6	52.6 ± 11.2	0.963
Gender (male/female)	36/24	32/28	0.58
BMI	23.51 ± 2.31	23.40 ± 2.64	0.808
CRP	6.76 ± 0.85	97.54 ± 27.14	0.001
PCT	0.07 ± 0.03	8.87 ± 1.24	0.001
BNP	45.40 ± 8.6	270.6 ± 24.6	0.001

BMI, body mass index; CRP, C-reactive protein; PCT, procalcitoninl; BNP, Brain Natriuretic Peptide

### Clinical value of miR-499a-5p level in SIMD patients

**Fig. 2:**
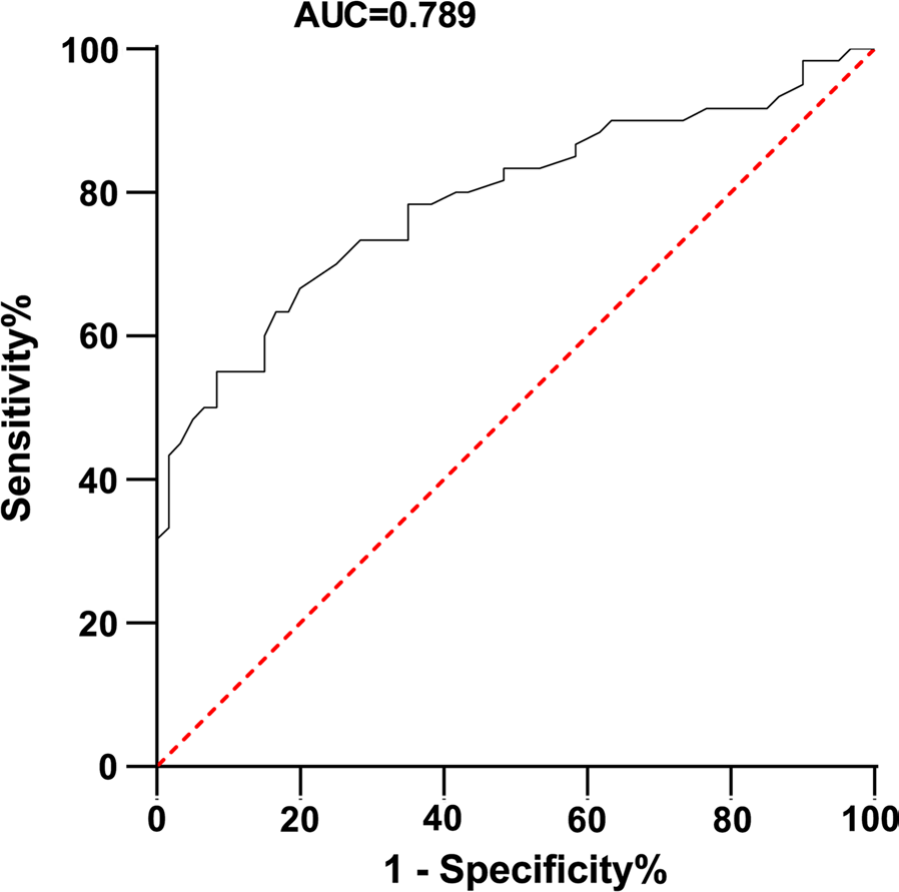
Clinical value of miR-499a-5p level in SIMD patients. The diagnostic value of miR-499a-5p in sepsis was analyzed by the ROC curve. The AUC was 0.789. When the critical value was 0.885, the sensitivity was 66.67% and the specificity was 80.0%

The diagnostic value of mir-499a-5p in sepsis myocardial dysfunction was determined by plotting ROC curve. As shown in Fig. 2, when the cutoff value was 0.885, the corresponding AUC value was 0.789, the sensitivity was 66.67%, and the specificity was 80.0%. Next, we evaluated the association between miR-499a-5p and the prognosis of SIMD patients. As shown in Table 3, a logistic regression analysis showed that the miR-499a-5p expression pattern (OR = 3.28, 95% CI 1.09–9.95, *P* < 0.05) and BNP expression pattern (OR = 2.95, 95% CI 1.01–8.60, *P* < 0.05) served as independent prognostic factors for 28-day survival in patients with sepsis. Briefly, miR-499a-5p elicited good diagnostic and prognostic value for SIMD.

**Table 3:** Correlation between different variables and survival time of patients with sepsis myocardial dysfunction

Variables	OR	95%CI	*P* value
*miR-499a-5p*	3.14	1.07–9.27	0.03
Age	0.61	0.21–1.75	0.42
Gender	0.81	0.28–2.32	0.79
BMI	0.97	0.34–2.79	0.95
CRP	1.44	0.50–4.18	0.5
PCT	1.08	0.38–3.10	0.89
BNP	2.95	0.97–8.89	0.04

OR, Odds ratio; CI, confidence interval; BMI, body mass index; CRP, C-reactive protein; PCT, procalcitonin

### miR-499a-5p upregulation alleviates SIMD

**Fig. 3:**
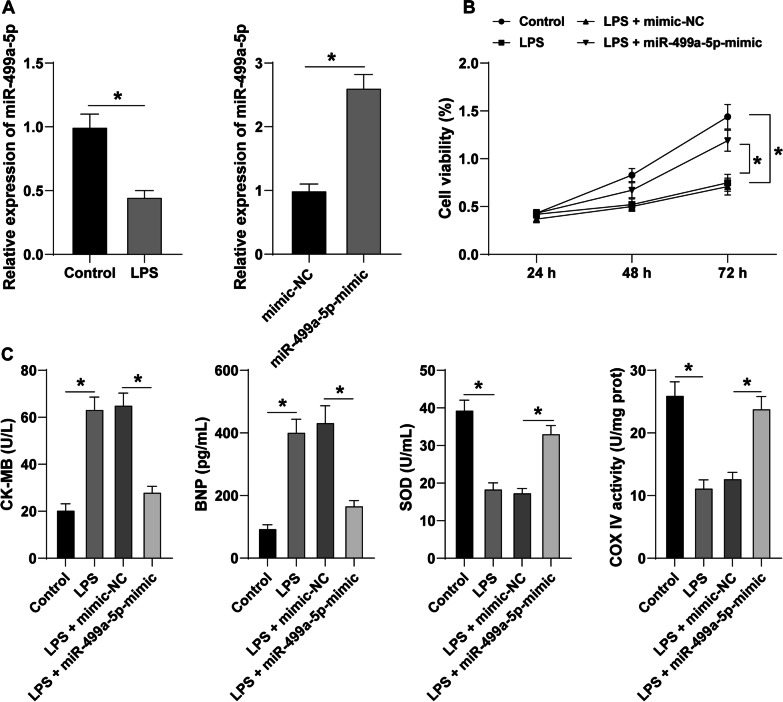
Upregulation of miR-499a-5p reduced the myocardial dysfunction in sepsis. AC16 cells were transfected with mimic-NC or miR-499a-5p-mimic, followed by LPS treatment. **a** The expression of miR-499a-5p was detected by RT-qPCR; **b** CCK-8 assay was used to detect cell viability; **c** the expression of CK-MB, BNP, SOD and COX IV were detected by ELISA; Three independent repeated tests were carried out, and the data were depicted as mean ± SD. One-way ANOVA was used for data analysis in panels **a**/**c**, two-way ANOVA was used for data analysis in panel **b**, and Tukey's post hoc test was used for post hoc test. *P < 0.05

To investigate the action of miR-499a-5p in SIMD in vitro, the miR-499a-5p-mimic was delivered into the AC16 cells. RT-qPCR was conducted to determine the expression pattern of miR-499-5p in AC16 cells. The results showed that the miR-499a-5p expression pattern in LPS group was lower than the expression pattern in control group; miR-499a-5p in AC16 cells after transfection of miR-499a-5p-mimic had evidently increased (all *P* < 0.05) (Fig. 3a). Subsequent CCK-8 assay (Fig. 3b) revealed that compared with the control group, the cell viability of LPS group was significantly limited; compared with LPS + mimic-NC group, the cell viability was considerably higher in the LPS + miR-499a-5p-mimic group (all *P* < 0.05). ELISA (Fig. 3c) revealed that the CK-MB and BNP concentrations of the LPS group was significantly elevated while the concentrations of SOD and COX IV were reduced; however miR-499a-5p overexpression could annul their levels (all *P* < 0.05).

### EIF4E is a target of miR-499a-5p

**Fig. 4:**
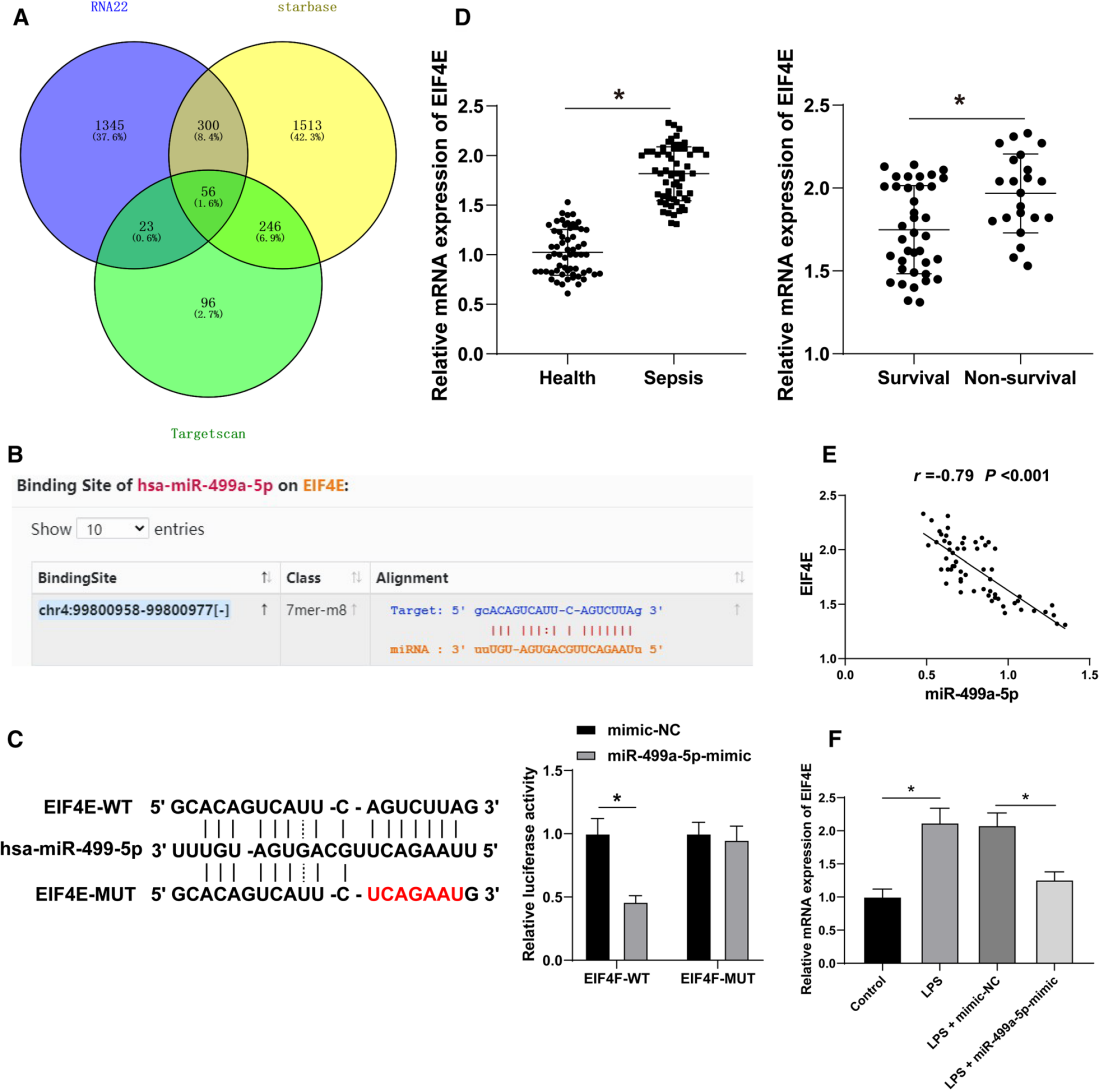
EIF4E is the downstream target of miR-499a-5p. **a** Through the RNA22, Starbase and TargetScan, the target genes of miR-499a-5p were screened by Venny map; **b** the binding sites of miR-499a-5p and EIF4E were predicted by the Starbase; **c** dual-luciferase assay verified that miR-499a-5p combined with EIF4E; **d** the expression of EIF4E mRNA in sepsis patients and healthy volunteers, survival group and non-survival group was detected by RT-qPCR (N = 60); **e** the correlation between miR-499a-5p and EIF4E was analyzed; **f** the expression of EIF4E mRNA in AC16 cells was detected by RT-qPCR; The data of **c** and **f** were depicted as mean ± SD. Two-way ANOVA was used in figure **c** and one-way ANOVA was used in figure **f**, followed by Tukey's post hoc test, and independent t test was used in figure **d**. *P < 0.05

Next, we investigated the downstream target genes of miR-499a-5p to study its regulatory mechanism. A combination online analysis of RNA22, Starbase and TargetScan predicted the downstream targets of miR-499a-5p and further identified the intersection (Fig. 4a), from which we focused on EIF4E, which is of vital significance in septic myocardial dysfunction [17]. The binding sites between miR-499a-5p and EIF4E were predicted by Starbase (Fig. 4b). Dual-luciferase assay identified a targeted relationship between miR-449a-5p and EIF4E. RT-qPCR showed that EIF4E mRNA in serum of SIMD patients was higher compared to the healthy controls, while the EIF4E mRNA in the survival group was significantly lower compared to the non-survival group (Fig. 4d). Correlation analysis identified a negative correlation between miR-499a-5p and EIF4E (R = −0.79, *P* < 0.001) (Fig. 4e). RT-qPCR showed that the EIF4E mRNA in the AC16 cells of the LPS group was higher relative to the control group; compared with the LPS + mimic-NC group, the EIF4E mRNA in the LPS + miR-499a-5p group was significantly lowered (all *P* < 0.05) (Fig. 4f).

### EIF4E overexpression annuls the protection of miR-499a-5p on SIMD

**Fig. 5:**
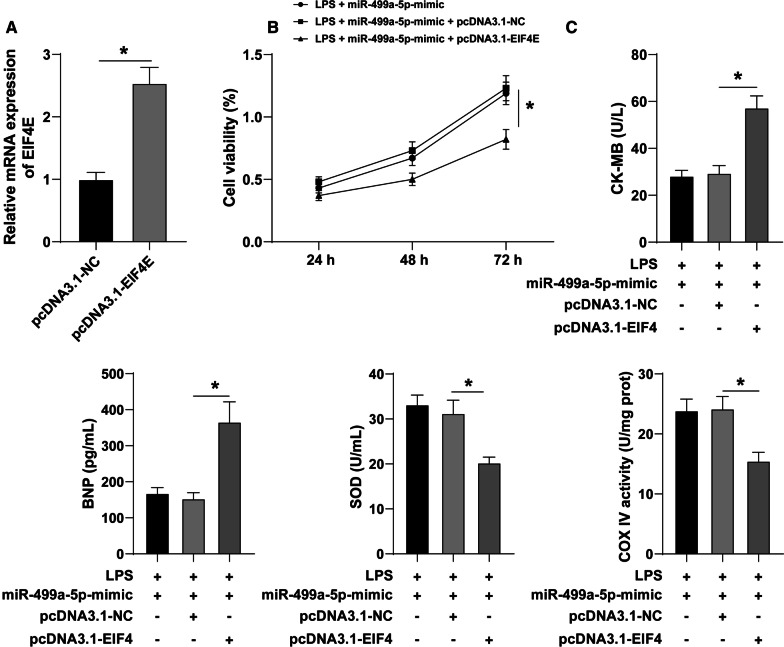
Overexpression of EIF4E reverses the role of miR-499-5p in SIMD. AC16 cells treated with LPS and transfected with miR-499a-5p-mimic were transfected with oe-NC or oe-EIF4E. **a** The expression of eIF4E mRNA was detected by RT-qPCR; **b** CCK-8 was used to detect cell viability; **c** the expressions of CK-MB, BNP, SOD and COX IV were detected by ELISA. Three independent repeated experiments were carried out, and the data were depicted as mean ± SD. T test was used for data analysis in panel **a**, two-way ANOVA was used for panel **b**, and one-way ANOVA was used for panel **c**, followed by Tukey’s post hoc test. *P < 0.05

Next, we sought to determine the involvement of EIF4E in the regulation of miR-499-5p on SIMD. The EIF4E mRNA was elevated in the AC16 cells after transfection of oe-EIF4E group (*P* < 0.05) (Fig. 5a). CCK-8 assay revealed that (Fig. 5b) compared with the LPS + miR-499a-5p + oe-NC group, AC16 cell viability in the LPS + miR-499a-5p + oe-EIF4E group was evidently decreased (*P* < 0.05). ELISA (Fig. 5c) showed that the concentrations of CK-MB and BNP were noticeably elevated, while the concentrations of SOD and COX IV were reduced after upregulating EIF4E (*P* < 0.05). These results indicate that miR-499-5p can regulate SIMD by targeting EIF4E.

## Discussion

SIMD has been regarded as a major complication of sepsis [27]. Fundamentally, the miR-499a is profusely expressed in adult cardiomyocytes with vital functionality in cardiac differentiation [28]. The current study explored the diagnostic and prognostic value of miR-499a-5p on SIMD.

Firstly, an extensive analysis of the clinical characteristics and miRNA expressions of SIMD patients and healthy controls was conducted. PCT is a polypeptide indicated in sepsis for the identification of non-systemic inflammatory response syndrome and CRP is a terminal biomarker for differentiating between inflammatory response and sepsis [29]. An existing study determined an association between BNP and the systolic and diastolic performance of left ventricular and right ventricular dysfunction in septic cardiomyopathy [30]. Our results presented with elevated expressions of serum PCT, CRP and BNP in SIMD patients relative to that in healthy controls. Liu et al. revealed the down-regulation of miR-499a-5p in myocardial ischemia/reperfusion (I/R) injury [31]. Additionally, our findings denoted a weak expression of miR-499a-5p in SIMD patients while no significant difference was evident in the expressions of miR-451 and miR-378 showed. Moreover, a higher miR-499a-5p expression was determined in the survival group of SIMD patients relative to the non-survival group. A recent study elucidated a poor expression of miR-499a-5p in H/R myocardial cell injury [16]. These results implicated a potential association between miR-499a-5p and the occurrence and prognosis of SIMD. Furthermore, we sought to evaluate the correlation of miR-499a-5p with SIMD to validate the diagnostic and prognostic value of miR-499a-5p in SIMD. Our results showed that AUC of miR-499a-5p was 0.789 with 66.67% sensitivity and 80.0% specificity at the cut-off value of 0.885, and implicated miR-499a-5p and BNP as independent prognostic factors for 28-day survival in sepsis patients, which was exhibitive of the high diagnostic and prognostic values of miR-499a-5p on SIMD.

Next, the miR-499a-5p-mimic was transfected into AC16 cells to investigate the effect of miR-499a-5p on SIMD in vitro. An existing study elucidated the ability of miR-499a overexpression to exacerbate the differentiation of human bone marrow-derived mesenchymal stem cells into cardiomyocytes with improved cardiomyogenesis [28]. Our results revealed a lowered miR-499a-5p expression in LPS-treated AC16 cells and an increased expression in AC16 cells treated with LPS and miR-499a-5p-mimic. miR-499 knockdown decreases cell viability in cardiomyocyte [32]. Our results showed decreased cell viability in LPS-treated AC16 cells, while it was increased in AC16 cells treated with LPS and miR-499a-5p-mimic. Consistently, after overexpression of miR-499a-5p, H9c2 cell apoptosis and LDH activity were decreased, thus eliciting the capacity of miR-499a-5p to alleviate H/R-induced cardiomyocyte injury [16]. CK-MB is a cardiac biomarker [33]. Oxidative stress marker SOD and mitochondrial function marker Cox IV are related to myocardial protection [34–36]. Our result presented with elevated concentrations of CK-MB and BNP while the concentrations of SOD and COX IV were reduced in LPS-treated AC16 cells, whereas an overexpression of miR-499a-5p could invert the findings. Shan et al. have implicated miR-499a-5p upregulation in the mitigation of cerebral I/R injury [37]. Similarly, our result elicited that miR-499a-5p overexpression alleviated SIMD.

The downstream mechanism of miR-499a-5p in SIMD was explored subsequently. The downstream targets were predicted, after which a correlation was identified between EIF4E phosphorylation and myocardial I/R recovery [38]. The binding sites of miR-499a-5p and EIF4E were predicted on StarBase database and their target relation was verified by dual-luciferase assay. Previously, sepsis has been implicated in the reduction of EIF4E phosphorylation [39]. Our result presented with an increased mRNA expression of EIF4E in sepsis patients and LPS-induced AC16 cells, while the mRNA expression was decreased in LPS-treated AC16 cells after miR-499a-5p mimic treatment, thus validating EIF4E as the downstream target of miR-499a-5p. An existing study demonstrated that EIF4E knockdown inhibits the extensive proliferation and migration of airway smooth muscle cells induced by platelet-derived growth factor BB in asthma [40]. To validate the involvement of EIF4E in SIMD regulated by miR-499a-5p, EIF4E was overexpressed in AC16 cells. Our results presented with decreased AC16 cell viability after treatment with LPS, miR-499a-5p and EIF4E overexpression. An existing study highlighted the role of eIF4E-binding protein 1 (4E-BP1) as a modulator of cardiomyocyte viability [41]. Our results showed EIF4E overexpression increased the concentrations of CK-MB and BNP while it simultaneously reduced the concentrations of SOD and COX IV in AC16 cells. Sulforaphane downregulates BNP level and represses EIF4E phosphorylation in H9c2 cells [42]. Therefore, our results suggested that miR-499a-5p could regulate SIMD via EIF4E.

## Conclusions

To conclude, the current study revealed the diagnostic and prognostic value of miR-499a-5p on SIMD, and preliminarily explored the underlying mechanism of miR-499a-5p in SIMD via EIF4E. Our results are indicative of the good diagnostic efficacy and predictive values of miR-499a-5p for SIMD, and further speculate its clinical diagnostic significance in surgical intervention. However, whether the serum miR-499a-5p level has the same diagnostic efficacy and predictive value in other diseases is uncertain, especially myocardial related diseases. More exploration is required. The in vitro mechanism of miR-499a-5p in myocardial dysfunction was explored, however verification by in vivo experiment is warranted. The effect of miR-499a-5p on other aspect of SIMD, such as, inflammation and cardiac function was not studied. Moreover, the clinical samples were limited to 60 for the analysis of clinical features and prognosis, and only 1 downstream target gene of miR-499a-5p was investigated in the current study. Thus, further investigation is warranted with an in vivo model, a bigger patient sample, more miRNAs and their target genes for comprehensive treatment of SIMD.

## Data Availability

The data and materials that support this study are available from the corresponding author upon reasonable request.

## Abbreviations

SIMD: Sepsis-induced myocardial dysfunction
miRNAs: MicroRNAs
CK-MB: Creatine kinase-MB isoform
BNP: Brain natriuretic peptide
H/R: Hypoxia/reoxygenation
EIF4E: Eukaryotic translation initiation factor 4E
CRP: C-reactive protein
PCT: Procalcitonin
FBS: Fetal bovine serum
LPS: Lipopolysaccharide
RT-qPCR: Reverse transcription quantitative polymerase chain reaction
CCK-8: Cell counting kit-8
ELISA: Enzyme-linked immunosorbent assay
ANOVA: Analysis of variance
ROC: Receiver operating characteristic
I/R: Ischemia/reperfusion
4E-BP1: EIF4E-binding protein 1
